# Assessment of electrocardiogram abnormality and associated factors among apparently healthy adult type 2 diabetic patients on follow-up at Jimma Medical Center, Southwest Ethiopia: Cross-sectional study

**DOI:** 10.1186/s12872-021-02110-6

**Published:** 2021-06-24

**Authors:** Deriba A. Bedane, Samuel Tadesse, Moyeta Bariso, Wondu Reta, Gaddisa Desu

**Affiliations:** 1 Department of Biomedical Sciences, Physiology Unit, College of Medical Sciences, Institute of Health, Jimma University, Jimma, Ethiopia; 2 Jimma Medical Center, Department of Internal Medicine, College of Medical Sciences, Institute of Health, Jimma University, Jimma, Ethiopia

**Keywords:** Type 2 diabetes mellitus, ECG abnormality, Minnesota ECG criteria, Jimma medical center

## Abstract

**Background:**

Diabetes mellitus is a group of metabolic disorders causing long-term damage to the cardiovascular system which remains asymptomatic among diabetic patients. An electrocardiograph is a simple and first-line tool in the screening of cardiovascular diseases.

**Objective:**

To assess electrocardiogram abnormality and associated factors among apparently healthy adult type 2 diabetes patients on follow-up at Jimma Medical Center, 2019.

**Materials and methods:**

Institutional based cross-sectional study was conducted from April 1 to May 30, 2019, at Jimma Medical Center among selected type 2 diabetes patients. Systematic random sampling was employed to select the study participants. The World Health Organization stepwise approach and interviewer-administered semi-structured questionnaires were employed to collect basic data. Resting Electrocardiography was done using a standard 12-lead electrocardiograph machine. The collected data were checked for completeness, coded, entered into the Epi-data Version 4.0.2. and exported to SPSS Version 21. Descriptive statistics like frequencies, percentages, mean and standard deviations were carried out. Binary and multiple logistic regression was done and a *p* value of less than 0.05 was used as a level of significance.

**Results:**

A total of 344 type 2 diabetes patients were interviewed and underwent electrocardiography making a 100% response rate. Electrocardiographic abnormality was identified among 209 (61%) of the respondents. Not attending formal education [AOR = 3.07, 95%, CI = 1.37–6.87], solid oil use, [AOR = 1.79, 95%, CI = 1.07–2.98], body mass index ≥ 25 kg/m^2^ [AOR = 2.74, 95%, CI = 1.67–4.50] and long duration of diabetes ≥ 10 years [AOR = 3.36, 95%, CI = 1.46–7.71] were associated with electrocardiogram abnormality.

**Conclusions:**

and recommendation

In this study, the majority (3/5th) of the participant had electrocardiogram abnormality. Not attending formal education, longer duration of diabetes ≥ 10 years, solid oil use, and increased body mass index ≥ 25 kg/m^2^ were independent predictors of electrocardiographic abnormality. Integrating electrocardiogram screening in routine diabetic management can pick cardiac complications of diabetes.

**Supplementary Information:**

The online version contains supplementary material available at 10.1186/s12872-021-02110-6.

## Background

Diabetes mellitus (DM) is a group of metabolic disorders characterized by chronic hyperglycemia due to defects in insulin secretion, action, or both which leads to metabolic disturbances [1]. Type 2 diabetes mellitus (T2DM) is caused by a progressive loss of β-cell insulin secretion on the background of insulin resistance [2].

Diabetes mellitus is growing globally at a rapid rate especially in middle and low-income countries like Sub-Sahara aggravated by a change in socio-economic, nutritional, and lifestyles [3]. There were about 451 million diabetic adults between 18–99 years in 2017 making the global adult prevalence of 8.8%. The adult prevalence of diabetes was 4.2% in Africa and 5.2% in Ethiopia in 2015 [4]. Currently, an epidemic of T2DM is increasing worldwide with 80% of them are living in low to middle-income countries [5]. In Africa, T2DM accounts for about 90–95% of all diabetes [6]. About 49.7% of the world and 69.2% of Africa's diabetes remained undiagnosed [7]. T2DM may present with or without symptoms and might cause long-term damage to the cardiovascular system [8].

Diabetes and CVDs account for more than 80% of deaths in developing countries [9]. The leading causes of morbidity and mortality among T2DM are atherosclerotic cardiovascular diseases such as coronary heart disease, cerebrovascular disease, or peripheral arterial disease [5]. There were about 3.7 million deaths due to hyperglycemia in 2012 worldwide. From these, 1.5 million deaths due to DM while 2.2 million CVDs deaths [10]. CVDs were the 2nd while diabetes was the 9th leading cause of premature death and disability in Ethiopia [11].

A link between DM and cardiovascular diseases (CVDs) is a central cause of morbidity and mortality in diabetic patients [12]. The relationship between diabetes and CVDs is complex and multifactorial including autonomic dysfunction, atrial and ventricular remodeling, and molecular alterations [13]. Additionally, dysglycemia, dyslipidemia, and hyperinsulinemia change metabolic profiles and cellular signaling of the cardiovascular system [14]. These all increase the risks of CVDs among diabetic populations. American heart association states CVDs risks increased 2- 4 times among T2DM [15].

Electrocardiogram (ECG) is the recording of cardiac electrical activity which provides the duration and amount of electrical activity of heart muscles [16]. A resting 12-lead electrocardiogram is frequently used in evaluating patients with suspected cardiovascular disease [17]. Usually, physicians do not routinely screen diabetic patients for arteriosclerotic unless the disease is suspected [18]. The presence of major ECG abnormality is associated with an increased risk of CVDs in diabetic patients [19]. To our knowledge, there is no study conducted in Ethiopia to assess the ECG abnormality and associated factors among an asymptomatic diabetic patient. Implementing an efficient noninvasive screening and identification of cardiac abnormalities can pick unrecognized and asymptomatic diabetic cardiac impairment [20]. Thus, this study is aimed to assess electrocardiogram abnormality and associated factors among apparently healthy adult T2DM patients.

## Methods

### Study design and setting

An Institutional based cross-sectional study was conducted from April, 1 to May 30, 2019, at Jimma Medical Center (JMC) which is located in Oromia Regional State, 350 km southwest of Addis Ababa. JMC is the referral teaching hospital giving health services to about 15 million people in the South West of Ethiopia. Diabetic patients getting follow-up service in the diabetic clinic are usually appointed in one to two months regularly.

### Study population and eligibility

All adult type 2 diabetic patients who were on follow-up at diabetic clinics at JMC were a source population. The study population was all randomly selected adult type 2 diabetic patients on follow-up at JMC during the study period and who fulfill the eligibility criteria. All type 2 diabetic patients aged 18 years and above, who had no prior diagnosis of cardiovascular diseases were included in this study. Type 2 diabetic patients who were mentally impaired and unable to give information, or had diagnosed CVDs before the onset of diabetes mellitus were excluded. Type 2 diabetic patients with concomitant diseases like chronic liver disease, kidney disease, thyroid diseases, etc., as well as type 2 diabetic with acute complications diabetes ketoacidosis, or hyperosmolar hyperglycemic state, were also excluded.

### Sample size and sampling procedure

The sample size was determined using a single population proportion formula by considering the following assumptions: *p* = 50% since no similar study done in Ethiopia, the margin of error = 5% and 95% confidence level $$n = \frac{{\left( {z{\raise0.7ex\hbox{$\alpha $} \!\mathord{\left/ {\vphantom {\alpha 2}}\right.\kern-\nulldelimiterspace} \!\lower0.7ex\hbox{$2$}}} \right)^{2} p\left( {1 - p} \right)}}{{d^{2} }} = \frac{{(1.96)^{2} \times 0.5 \times 0.5}}{{(0.05)^{2} }} = 384.$$ From chronic follow-up record data, the total number of type 2 diabetic patients on follow-up at JMC in 2018 was 1685. Since the source population is less than 10,000 applying the formula for finite population correction the final sample size was calculated as follows.

$nf = \frac{n}{{\{ 1 + (n/N\} }} = \frac{{384}}{{\{ 1 + (384/1685)\} }} = 313.$ Adding 10% non-response rate = 313 + 31 = 344.

### Data collection tools and procedure

World health organization (WHO) stepwise approach was employed for collecting the risks of CVDs among type 2 diabetic patients during entry [21]. The questionnaire was translated from English to Afan Oromo and back to English to assure its consistency in a blinded manner. A pre-test was done on 5% of the total sample size at Shanan Gibe Hospital before the actual data collection to ensure clarity, understandability, and completeness. Correction and modification on grammar, sequences, and timing were made based on the result of the pre-test before the start of actual data collection. Finally, the Afan Oromo version was used to collect data. Data collection was conducted through a face-to-face interview by trained data collectors using semi-structured questionnaires. The information related to the medical condition was reviewed from patients’ medical charts. The questionnaires were checked for completeness by supervisors and principal investigators every day.

### Study variables

The dependent variable of the study was ECG abnormality. The independent variables were sociodemographic factors (age, sex, educational status, occupation, and place of residence), factors related to a medical condition (duration of treatment and glycemic control), behavioral factors (physical activity, alcohol, tobacco, and khat use), nutritional factors (the type of cooking oil and fruit and vegetable intake), and factors related with body composition (weight, height, body mass index (BMI), waist circumference (WC), blood pressure (BP), and waist to hip ratio (WHR)).

### Operational definition

*Electrocardiography* Recording the heart’s electrical echoes using the ECG machine by placing electrodes on the surface of the body.

*Normal sinus rhythm* A regular heart rate between 50 and 100 beats per minute [22] with normal p waves, PR interval, QRS complex, T waves, and p waves preceding each QRS complex.

*ECG abnormality* Any ECG change beyond normal sinus rhythm (ST-segment elevation or depression, T- wave aberrations (inversion or tall T-wave), AV nodal block, bundle branch block, chamber enlargement, and dilatation, ventricular hypertrophy, arrhythmias, and prolonged QT intervals).

*Solid oil* The palm oil which is solid at room temperature.

### Measurements

The measurements of blood pressure, anthropometry (weight, height, waist circumference, and hip circumferences), fasting blood glucose level, and recording of electrocardiography were made according to the respective guidelines. Accordingly, the measurements of anthropometry were measured according to WHO guidelines [23] while BP was measured according to the European society of hypertension recommendation [24]. Fasting blood sugar (FBS) was measured according to Standard Operating Procedures Clinical and Translational Research Center [25] and ECG was recorded according to the standard manual for the Minnesota ECG criteria [22]. The height was measured to the nearest 0.5 cm with a stadiometer (Prestige Tokyo, Japan) as participants stand vertical with no shoes or headwear with their back against the stadiometer, heels together and eyes focused forward. The weight was measured to the nearest 0.1 kg while the participant wore light clothing with a digital weighing scale (Tanita Corporation, Tokyo, Japan) and checked daily with a known weight. WC was measured in a standing position using a non-stretchable measuring tape meter at the midpoint between the costal margin and iliac crests at the end of expiration to the nearest centimeter. HC was measured as the maximum circumference at the level of the greater trochanter (the widest portion of the hip) on both sides using a non-stretchable measuring tape meter. FBS was done with a simple finger prick using a digital glucometer. BP was measured three times in sitting position from the non-dominant arm at heart level using an aneroid sphygmomanometer (Yton sphygmomanometer, Italy) after the participant rested for 5 min before taking the measurement and 3 min before repeating the subsequent measurements, and the average was used for analysis. The resting 12 lead ECGs were obtained after a 10-min rest, with 10 mm/mV amplitude and paper speed of 25 mm/s rates with standard lead positions in a supine position using YORK” 12 lead Electrocardiography (India). The recorded ECG was coded according to the Minnesota code and manually read by two cardiologists in a blinded manner having no information about the patients. The suggested procedure for electrocardiogram was followed in the reference to the standard manual for the Minnesota ECG criteria [22].

### Data processing and analysis

The data were entered into Epi-data version 4.0.2. and exported to SPSS version 21 for analysis after checking for completeness. The exported data were explored to check outliers and missing values. Descriptive statistics like frequencies, percentages, mean, and standard deviations were carried out. Cross-tabulations and binary variable analyses were performed to select variables for multivariable analysis. The variables with a *p* value < 0.25 in the bivariable analysis were taken as candidates for multivariable analysis. Finally, multivariable logistic regression analysis was done using backward selection and the variables with a *p* value of less than 0.05 were taken as statistically significant determinants of abnormality of ECG. The odds ratio with its 95% CI was used to show the degree of association and estimation between the independent and the outcome variables.

## Results

### Socio-demographic characteristics

A total of 344 T2DM patients were interviewed and underwent electrocardiography giving a response rate of 100%. The majority of 210 (61%) of the respondents were males. The mean age of the respondents was 53.34 ± 11.07 years with a minimum age of 18 and a maximum of 80 years. More than half, 202 (58.7%) of the respondents were in the age group of 51–70 years. Farmer respondents were 109 (31.7%) while merchants and government employees accounted for 83 and 84 (24%) respectively. More than half 199 (57.8%) of the respondents were urban dwellers (Table 1).

**Table 1:** Sociodemographic characteristics of apparently healthy adult type 2 diabetes patients on follow-up at JMC May, 2019

Variable	Category	Frequency	Percent
Age group in years	< 40	46	13.4
	41–50	70	20.3
	51–60	111	32.3
	61–70	91	26.4
	> 70	26	7.6
Sex	Male	210	61.0
	Female	134	39.0
Occupational status	Farmer	109	31.7
	Daily laborer	12	3.5
	Merchant	83	24.1
	Government employee	84	24.4
	NGO/private	31	9.0
	Others^a^	25	7.3
Educational status	No formal education	63	18.3
	Primary education	136	39.5
	Secondary education	74	21.5
	Tertiary education	71	20.6
Place of residency	Urban	199	57.8
	Rural	145	42.2

^a^Housewife, students, pensions, carpenter

### Behavioral measurements of the respondents

The majority of the respondents, 333 (96.8%) and 319 (92.7%) were nonsmokers and non-alcohol users respectively. Most of the respondents 331 (96.2%) were involved in physical activity less than 150 min of moderate or 75 min of vigorous physical activity per week (less than 600 metabolic equivalents of energy per week). Non-khat chewers accounted for 304 (88.4%) of the respondents.

### Dietary factors of the respondents

Most of the respondents 339 (98.5%) were using fruits and vegetables below five servings per day. Five servings of fruit and vegetables per day (equivalent to 400 g of fruits and vegetables) while solid oil users were 235 (68.3%).

### Blood pressure measurements of the respondents

An average systolic blood pressure (SBP) was 121.20 ± 7.34 mmHg while the average diastolic blood pressure (DBP) was 77.85 ± 4.15 mmHg (Table 2).

**Table 2:** Distribution of anthropometric and blood pressure measurement of apparently healthy adult type 2 diabetes patients on follow-up at JMC May 2019

Variable	Category	Frequency	Percent
Systolic BP	< 120 mmHg	106	30.8
	120–139 mmHg	238	69.2
Diastolic BP	< 80 mmHg	161	46.8
	80–89 mmHg	183	53.2
BMI category	Underweight	27	7.8
	Normal weight	183	53.2
	Over weight	85	24.7
	Obese	49	14.2
WC category	Normal	126	36.6
	Increased risk	218	63.4
WHR	Normal	48	14.0
	Increased risk	296	86.0

### Duration of diabetes and blood glucose levels of the respondents

**Fig. 1:**
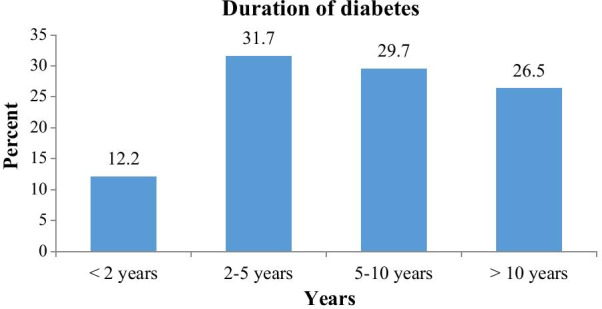
Duration of diabetes of apparently healthy adult type 2 diabetes on follow up at JMC May 2019

The mean fasting blood glucose was 123.65 ± 17.34 mg/dl. From the total respondents, 210 (61%) had a fasting blood sugar within a normal range whereas hyperglycemia and hypoglycemia were 123 (35.8%) and 11 (3.2%) respectively. The mean duration of diabetes was 7.31 ± 4.54 years with a minimum duration of 1 year and a maximum of 19 years. Most of the respondents 109 (31.7%) and 102 (29.7%) had a diabetes duration of 2–5 and 5–10 years respectively (Fig. 1).

### ECG status of the respondents

**Fig. 2:**
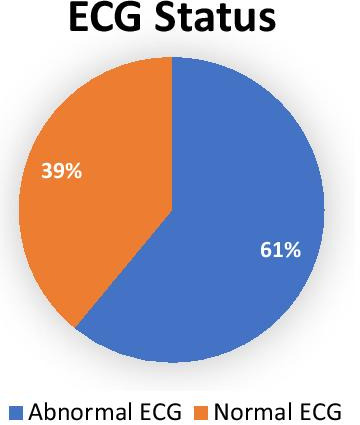
Normal and abnormal ECG status of apparently healthy adult type 2 diabetes on follow up at JMC May 2019

The majority of the respondents 209 (61%) had at least one electrocardiogram abnormality (Fig. 2). ECG abnormality was 101 (29.4%) among participants with abnormal body weight 21 (6.1%) among underweight, 57 (16.6%) among overweight, 23 (6.7%) among obese), 136 (39.5%) in high-risk WC, 69 (20.1%) in DM duration > 10 years, 158 (45.9%) in Solid oil users and 109 (31.7) urban dwellers.

### Factors associated with ECG abnormalities

#### Binary and multivariable analysis

Variables like age category, educational status, occupation, place of residence, duration of DM, solid oil use, BMI category, WC category, SBP, and fasting blood sugar were associated with ECG abnormalities at a *p* value of less than 0.25. The finding of multivariable logistic regression indicated that educational status, duration of DM, solid oil, and BMI category were independently associated with ECG abnormality.

Diabetic patients who had no formal education were 3 times higher odds of more likely having abnormal ECG compared to those who had attended above secondary education (AOR = 3.07, 95%, CI = 1.37–6.87). The odds of having ECG abnormality were 1.8 times higher among solid oil users compared to their counterparties (AOR = 1.79, 95%, CI = 1.07–2.98). ECG abnormality also showed association with participants with a BMI of ≥ 25 kg/m^2^. Patients with BMI ≥ 25 kg/m^2^ were 2.7 times odds more likely to develop ECG abnormality compared to normal weighted diabetic patients (AOR = 2.74, 95%, CI = 1.67–4.50). The duration of DM had also an association with ECG abnormalities. Those who had a duration of diabetes greater than 10 years were 3 times the odds more likely to develop ECG abnormality compared with patients with a duration of fewer than two years (AOR = 3.36, 95%, CI = 1.46–7.71) (Table 3).

**Table 3:** Bivariable and multivariable analysis of factors associated with ECG abnormalities among apparently healthy adult type 2 diabetes on follow-up at JMC May 2019

Variable	Category	ECG abnormality		COR (95% CI)	AOR (95% CI)
		No (%)	Yes (%)		
Age group (yrs.)	< 40	23 (6.7)	23 (6.7)	1	1
	40–50	31 (9.0)	39 (11.3)	1.26 [.60–2.65]	1.03 [.45–2.38]
	51–60	40 (11.6)	71 (20.6)	1.78 [.89–3.56]	1.42 [.64–3.15]
	61–70	35 (10.2)	56 (16.3)	1.60 [.78–3.27]	1.14 [.50–2.61]
	> 70	6 (1.7)	20 (5.8)	3.33 [1.13–9.82]*	1.90 [.58–6.21]
Educational status	No formal education	14 (4.1)	49 (14.2)	3.81 [1.79–8.10]*	3.07 [1.37–6.87]*
	Primary education	51 (14.5)	85 (24.7)	1.81 [1.02–3.24]*	1.79 [.96–3.31]
	Secondary education	33 (9.6)	41 (11.9)	1.35 [.70–2.60]	1.65 [.82–3.35]
	Tertiary education	37 (10.8)	34 (9.9)	1	1
Occupation	Farmer	34 (9.9)	75 (21.8)	1	1
	Daily labor	5 (1.5)	7 (2.0)	.64 [.19–2.14]	.77 [.18–3.23]
	Merchant	36 (10.5)	47 (13.7)	.59 [.33–1.07]	1.14 [.46–2.81]
	Government employ	39 (11.3)	45 (13.1)	.52 [.29-.94]*	. 1.54 [.57–4.19]
	NGO/private	14 (4.1)	17 (4.9)	.55 [.24–1.24]	1.72 [.61–4.85]
	Other	7 (2.0)	18 (5.2)	1.17 [.45–3.05]	1.42 [.42–4.74]
Place of residency	Urban	90 (26.2)	109 (31.7)	.55 [.35-.85]*	.83 [.49–1.44]
	Rural	45 (13.1)	100 (29.1)	1	1
Solid oil use	Yes	77 (22.4)	158 (45.9)	2.33 [1.47–3.71]**	1.79 [1.07–2.98]*
	No	58 (16.9)	51 (14.8)	1	1
Duration of DM	< 2 years	20 (5.8)	22 (6.4)	1	1
	2–5 years	41 (11.9)	68 (19.8)	1.51 [.74–3.09]	1.79 [.83–3.88]
	5–10 years	52 (15.1)	50 (14.5)	.87 [.43–1.80]	1.02 [.47–2.21]
	> 10 years	22 (6.4)	69 (20.1)	2.85 [1.32–6.17]*	3.36 [1.46–7.71]*
Fasting BGL	Normal fasting BGL	91 (26.5)	120 (34.9)	1	1
	Abnormal fasting BGL	44 (12.8)	89 (25.9)	1.53 [.98–2.41]	1.43 [.86–2.37]
Average SBP	Normal BP	36 (10.5)	70 (20.3)	1	1
	Abnormal BP	99 (28.8)	139 (40.4)	.72 [.45–1.16]	.59 [.35–1.01]
WC category	Normal	53 (15.4)	73 (21.2)	**1**	**1**
	Risk	82 (23.8)	136 (39.5)	1.20 [.77–1.88]	1.19 [.70–2.03]
BMI category	Normal weight	90 (26.2)	94 (27.3)	1	1
	Abnormal weight	45 (13.1)	115 (33.4)	2.43 [1.55–3.82]**	2.74 [1.67–4.50]**

The bold is used to help the reader to easily get the variables that are significantly associated with ECG abnormality either in binary or multiple logistic regression

*p < 0.05; **p < 0.001

## Discussion

Two hundred nine (61%) of the respondents had at least one type of ECG abnormality. This finding is comparable with a study conducted in the United States (60%) [26]. But, it is lower than the study done in Uganda (67.8%) [27]. On the other hand, the present finding was higher than the studies conducted in Slovakia (53.7%) [28], India (26%) [29], and Sudan (23%) [30]. These differences may be due to the difference in the socio-economic, health care system, other co-morbidities, study design, selection criteria, the presence or absence of other risk factors, environmental and genetic variations.

Lack of formal education was one of the predictors of ECG abnormality among T2DM patients. This finding was supported by the study conducted in Sweden [31], Iran [32], Malaysia [33], and Japan [34] that revealed higher risks of CVDs among diabetic patients with low educational status. A study conducted in 20 developing countries showed that CVDs were more common among those with low levels of education [35]. The lack of education affects individuals’ health literacy. Inadequate health literacy might increase the risk of diabetic cardiac impairment [36].

The use of solid oil was one of the predictors of electrocardiographic abnormalities among T2DM patients in this study. High saturated fatty acid causes cardiometabolic dysfunction and poor glycemic control [37]. The cell membrane fatty acid change has a direct and indirect effect on the electrophysiological property [38]. This finding was in harmony with studies conducted in Europe [39] and Israel [40] that showed the substitution of carbohydrates with saturated fatty acid increases the risk of CVDs and as dietary modification reduces CVDs the risks among T2DM respectively. Saturated fatty acid use reduces endothelial function and insulin sensitivity being a risk of CVDs [41].

This study also revealed that increased body weight was also associated with ECG abnormality. An increment in body mass index greater than 25 kg/m^2^ was strongly associated with ECG abnormality. This finding was supported by the study conducted in Turkey on ECG parameter changes in overweight individuals compared with the normoweight [42]. Obesity alters the morphology and electrophysiology of myocardial cells and increases CVDs risks shifts the cardiac axis leftward [43] and may increase the risk of atherogenesis [44]. The meta-analysis findings revealed that excess weight is associated with CVDs mortality [45]. Obesity may also cause abnormal myocardial perfusion among T2DM patients [46].

The duration of diabetes greater than ten years was also among the factors that affect ECG patterns. This finding was supported by the study conducted in India showed the duration of diabetes mellitus 5–10 years had ECG changes [29]. Another survey conducted in Denmark also showed heart failure was diagnosed 37.4% after 10 years or more in type 2 diabetes [47]. Similarly, the study conducted in Sweden among diabetic patients revealed a longer duration of diabetes increases the risk of CVDs [48].

## Limitation of the study

Biochemical measurements like lipid profile and serum electrolytes were not done due to budget shortage.

## Conclusion and recommendation

In this study, the majority (3/5th) of the participant had ECG abnormality. Not attending formal education, longer duration of DM ≥ 10 years, solid oil use, and increased BMI ≥ 25 kg/m^2^ were independent predictors of ECG abnormality. Integrating ECG screening in routine diabetic management helps to better manage the impact of T2DM on the cardiovascular system.

## Supplementary Information

**Additional file 1**. The measurement procedures of blood pressure, anthropometry, fasting blood glucose level, and recording of electrocardiography.

**Additional file 2**. Patient’s information sheets.

## Data Availability

The datasets used and analyzed during the current study are available from the corresponding author on reasonable request.

## Abbreviations

AOR: Adjusted odds ratio
BMI: Body mass index
BP: Blood pressure
COR: Crude odd ratio
CVDs: Cardiovascular diseases
DM: Diabetes mellitus
ECG: Electrocardiogram
FBS: Fasting blood sugar
JMC: Jimma Medical Center
T2DM: Type 2 diabetes mellitus
WC: Waist circumference
WHO: World health organization
WHR: Waist to hip ratio
