# Supplementary material for: Assessment of electrocardiogram abnormality and associated factors among apparently healthy adult type 2 diabetic patients on follow-up at Jimma Medical Center, Southwest Ethiopia: Cross-sectional study
Source: BMC Cardiovasc Disord. 2021 Jun 24;21:312. doi: 10.1186/s12872-021-02110-6 (PMC8223340; doi:10.1186/s12872-021-02110-6)
Supplement: Supplementary file 1 — Additional file 1. The measurement procedures of blood pressure, anthropometry, fasting blood glucose level, and recording of electrocardiography. [file 12872_2021_2110_MOESM1_ESM.doc]

**Additinal file 1. doc. the measurement procedures of blood pressure, anthropometry, fasting blood glucose level, and recording of electrocardiography.**

The measurements of blood pressure, anthropometry (weight, height, waist circumference, and hip circumferences), fasting blood glucose level, and recording of electrocardiography were made according to the respective guideline as follow.

**Additional file 1A. doc. Blood pressure measurements procudures.**

Blood pressure was measured according to the European society of hypertension recommendation . BP was measured three times in sitting position from the non-dominant arm at heart level using an aneroid sphygmomanometer (Yton sphygmomanometer, Italy) after the participant rested for 5 minutes before taking the measurement and 3 minutes before repeating the subsequent measurements, and the average was used for analysis. The patient’s arm was rested comfortably with palm up and supported at the heart level with legs uncrossed. The patients sat relaxed and comfortably. The mean of the measurements was used for analysis.

**Additional fole 1B. doc. Anthropometric measurements (weight, height, waist circumference, and hip circumferences)**

The measurements of anthropometric measurements (weight, height, waist circumference, and hip circumferences) were measured according to the world health organization standard guidelines .
**Height:** The height was measured to the nearest 0.5cm with a stadiometer (Prestige Tokyo, Japan) as participants stand vertical with no shoes or headwear with their back against the stadiometer, heels together and eyes focused forward.

**Weight**: The weight was measured to the nearest 0.1kg while the participant wore light clothing with a digital weighing scale (Tanita Corporation, Tokyo, Japan) and checked daily with a known weight.
**Body mass index:** BMI was calculated using the formula: observed weight in kg divided by height in meter squared (kg/m²).
**Waist circumference:** waist circumference was measured in standing position using a non-stretchable measuring tape meter at the midpoint between the costal margin and iliac crests at the end of expiration to the nearest centimeter. All the participants were stood upright in a comfortable position with both feet together on a horizontal surface.
**Hip circumference:** Hip circumference was measured as the maximum circumference at the level of the greater trochanter (the widest portion of the hip) on both sides using a non-stretchable measuring tape meter. Measurements were noted to the nearest centimeter.
**Waist and hip ratio:** Waist circumference to hip ratio was obtained by dividing the waist circumference in cm by the hip circumference in centimeter.

**Aditional file 1C. doc. Fasting blood glucose level measurements of type 2 diabetes mellitus**

Fasting blood sugar was done with a simple finger prick using a digital glucometer. The patient’s finger was cleaned off with an alcohol pad, then dried with a clean gauze pad, and prepared for fingerpick. The finger test site was pricked with a quick motion and gently pressed and obtained a drop of blood at least one microliter. The drop of blood was held to a narrow channel in the test strip and blood was drawn into the strip. The blood glucose level was displayed along with the unit of measure and the result was recorded.

**Additinal file 1D. doc. Procedures and patient preparation for ECG recording of type2 diabetes mellitus**

The suggested procedure for obtaining a resting ECG and the technical requirements for a suitable ECG was followed in the reference to standard manual for the Minnesota code
**Equipment:** “YORK” 12 lead Electrocardiography manufactured by York Scientific company. The ECGs were obtained after a 10-minute rest, with 10 mm/mV amplitude and paper speed of 25 mm/s rate with standard lead positions in a supine position using YORK” 12 lead Electrocardiography (India). The recorded ECG was coded according to the Minnesota code and manually read by two cardiologists in a blinded manner having no information about the patients. The suggested procedure for electrocardiogram was followed in the reference to the standard manual for the Minnesota ECG criteria .
**Equipment:** “YORK” 12 lead Electrocardiography manufactured by York Scientific company.
**Calibration:** The paper speed is 25 mm/s and the voltage is 10 mm/mV.

**Patient preparation:** Patient preparation as well as the lead attachment was made according to the standard manual for Minnesota code protocol.

1. Hands were washed and the following equipment and supplies were gathered.

- 12-lead ECG Machine
- ECG Electrodes
- Alcohol swabs
- Adhesive remover swabs
- Gauze pads
- Surgical blade
- Cardiac jell

1. An ECG recorder was introduced him/herself and verified the patient’s name and chart.
2. The patient removed metallic ornaments and clothing from the waist up and ensuring privacy.
3. The ECG procedures were explained to the patients and answered their questions appropriately.
4. The patient was in a supine position and the skin was prepared for the placement of the electrode by shaving hair, rubbing the lower legs, lower forearms, and chest area with alcohol swabs, and dried the areas with gauze pads.
5. The electrodes were applied to start with the lower legs, lower forearms, and chest area.

**Electrode placement:**

1. Bipolar limb leads (frontal plane)
   1. Lead I: RA (-) to LA (+) (Anterolateral)
   2. Lead II: RA (-) to LL (+) (Inferior)
   3. Lead III: LA (-) to LL (+) (Inferior)
2. Augmented unipolar limb leads (frontal plane)
   1. Lead aVR: RA (+) to [LA & LL] (-) (Rightward)
   2. Lead aVL: LA (+) to [RA & LL] (-) (Anterolateral)
   3. Lead aVF: LL (+) to [RA & LA] (-) (Inferior)
3. Unipolar (+) chest leads (horizontal plane)
   1. Leads V1, V2, V3, V4 & V5: (Anterior)
   2. Lead V6: (Anterolateral)
4. V1: In the fourth intercostal space (between ribs 4 & 5) just to the right of the sternum.
5. V2: In the fourth intercostal space (between ribs 4 & 5) just to the left of the sternum.
6. V3: Between leads V2 and V4.
7. V4: In the fifth intercostal space (between ribs 5 & 6) in the mid-clavicular line.
8. V5: Horizontally even with V4, in the anterior axillary line.
9. V6: Horizontally even with V4 and V5 in the midaxillary line.
10. The lead wires were connected to the corresponding electrodes first beginning with the right leg.
11. Electrical equipment was turned off during recording.
12. The machine was turned on and entered the participant’s information ID number and date.
13. The patient was asked to breathe normally, relaxed, remained comfortable, and calm in the quiet room.
14. Then the ECG record was printed when the satisfactory waves were acquired.
15. Lead wires, electrodes, and adhesive gel were removed from the participant.
16. The traced ECG papers were kept for authorized reading.
17. The recorded ECG papers were read and interpreted by the cardiologist.
