# Supplementary material for: Assessment of electrocardiogram abnormality and associated factors among apparently healthy adult type 2 diabetic patients on follow-up at Jimma Medical Center, Southwest Ethiopia: Cross-sectional study
Source: BMC Cardiovasc Disord. 2021 Jun 24;21:312. doi: 10.1186/s12872-021-02110-6 (PMC8223340; doi:10.1186/s12872-021-02110-6)
Supplement: Supplementary file 2 — Additional file 2. Patient’s information sheets. [file 12872_2021_2110_MOESM2_ESM.docx]

**Addtional file 2 doc. Patient’s information sheets**

**Title of Research**: Assessment of electrocardiogram abnormality and associated factors among apparently healthy adult type 2 diabetes on follow up at Jimma medical center, 2019.

**Institution**: Jimma University institute of health, department of biomedical sciences (Post Graduate Program)

**Name of sponsor**: Jimma University

**Purpose of the study:** The purpose of this study is to determine the magnitude of electrocardiographic abnormality and associated factors among apparently healthy adult type 2 diabetes on follow up at JMC, 2019

Electrocardiographic abnormalities are common in patients with diabetes. Early screening of asymptomatic diabetes patients for cardiovascular help diabetic patients to be screened early for cardiovascular diseases to prevent complications that might happen. By considering this implication I want to take a study in this area. Please read the following description of the study and ask any unclear points before you agree to participate.

**Duration:** It will take about 20 minutes to understand the objective of the study, respond to the questions, and undergo electrocardiography.

**Procedure:** Before the Procedure to be carried out first, you will be asked few questions about your socioeconomic and demographic factors, behavioral factors, measurement of blood pressure, height, weight, waist circumference, hip circumferences, fasting blood sugar and ECG recording will be done. The principal investigator will cover the cost of fasting blood sugar and ECG recordings.

**Risk:** It may cause minimal discomfort but, will not cause you any physiological, financial, and psychological harm. This study will pick cardiovascular disease at an early stage so that early intervention will be taken.

**Benefits:** Even if there is no direct payment for your participation in this study, the information you give will help you and your physician to know your cardiovascular system. This also helps other diabetic patients by recommendation cardiovascular system screening management in diabetic patients. If your results indicate the presence of ECG abnormality, you will be referred to a cardiac clinic for further management.

**Confidentiality:** The unique code will be given for the information you will give us and your ECG result so that your result will not be identified. Only the principal investigator and selected health professionals have access to your result and the result will be used for this study only with confidentiality.

**Voluntary Participation and Withdrawal from the Study:** Your participation will be completely based on your willingness and you have the right not to participate and withdraw from participating in the study at any time after giving your consent and start your participation. You can also jump any question if you do not wish to respond to that question. This decision will not affect your current or future medical care in the health facility.

**Contact information:** If you have any questions about this study, you can contact the principal investigator.

**Principal Investigator:** Deriba A. Bedane

**Mobile:** +251917634318

**E-mail:** [deribaabera@gmail.com](mailto:deribaabera@gmail.com)
